# Genetic Determinants in a Critical Domain of NS5A Correlate with Hepatocellular Carcinoma in Cirrhotic Patients Infected with HCV Genotype 1b

**DOI:** 10.3390/v13050743

**Published:** 2021-04-23

**Authors:** Mohammad Alkhatib, Velia Chiara Di Maio, Valentina De Murtas, Ennio Polilli, Martina Milana, Elisabetta Teti, Gianluca Fiorentino, Vincenza Calvaruso, Silvia Barbaliscia, Ada Bertoli, Rossana Scutari, Luca Carioti, Valeria Cento, Maria Mercedes Santoro, Alessandro Orro, Ivana Maida, Ilaria Lenci, Loredana Sarmati, Antonio Craxì, Caterina Pasquazzi, Giustino Parruti, Sergio Babudieri, Luciano Milanesi, Massimo Andreoni, Mario Angelico, Carlo Federico Perno, Francesca Ceccherini-Silberstein, Valentina Svicher, Romina Salpini, on behalf of HIRMA (Hepatocarcinoma Innovative Research MArkers) and Fondazione Vironet C (HCV Virology Italian Resistance Network)

**Affiliations:** 1 Department of Experimental Medicine, University of Rome “Tor Vergata”, 00133 Rome, Italy; mohammad--alkhatib@hotmail.com (M.A.); Di.Maio@med.uniroma2.it (V.C.D.M.); s.barbaliscia@gmail.com (S.B.); bertoli@uniroma2.it (A.B.); scutari.rossana@gmail.com (R.S.); luca.carioti@yahoo.com (L.C.); santormaria@gmail.com (M.M.S.); ceccherini@med.uniroma2.it (F.C.-S.); rsalpini@gmail.com (R.S.); 2 Department of Clinical and Experimental Medicine, University of Sassari, 07100 Sassari, Italy; vdmurtas@uniss.it (V.D.M.); imaida@uniss.it (I.M.); babuder@uniss.it (S.B.); 3 Infectious Diseases Unit, Pescara General Hospital, 65124 Pescara, Italy; e.polilli@libero.it (E.P.); parruti@tin.it (G.P.); 4 Hepatology Unit, University Hospital of Rome “Tor Vergata”, 00133 Rome, Italy; martinamilana@gmail.com (M.M.); ilaria.lenci@uniroma2.it (I.L.); angelico@med.uniroma2.it (M.A.); 5 Infectious Diseases Unit, University Hospital of Rome “Tor Vergata”, 00133 Rome, Italy; elisabetta.teti@gmail.com (E.T.); sarmati@med.uniroma2.it (L.S.); andreoni@uniroma2.it (M.A.); 6 Infectious Diseases Unit, Sant’Andrea Hospital—“Sapienza” University, 00189 Rome, Italy; gianlucafiorentino87@gmail.com (G.F.); pasquazzi@yahoo.it (C.P.); 7 Gastroenterology, “P. Giaccone” University Hospital, 90127 Palermo, Italy; vincenza.calvaruso@unipa.it (V.C.); antoniocraxi@gmail.com (A.C.); 8 Laboratory of Clinical Microbiology and Virology, Polyclinic Tor Vergata Foundation, 00133 Rome, Italy; 9 Department of Oncology and Hemato-Oncology, University of Milan, 20122 Milan, Italy; valeria.cento@unimi.it; 10 ITB-CNR, Institute of Biomedical Technologies, National Research Council of Italy, 20090 Milan, Italy; alessandro.orro@gmail.com (A.O.); luciano.milanesi@itb.cnr.it (L.M.); 11 Department of Diagnostic and Laboratory Medicine, IRCCS *Bambino Gesu’*, Pediatric Hospital, 60165 Rome, Italy; perno@uniroma2.it

**Keywords:** hepatitis C virus, hepatocellular carcinoma, cirrhosis, NS5A, genotype 1b, genetic variability

## Abstract

HCV is an important cause of hepatocellular carcinoma (HCC). HCV NS5A domain-1 interacts with cellular proteins inducing pro-oncogenic pathways. Thus, we explore genetic variations in NS5A domain-1 and their association with HCC, by analyzing 188 NS5A sequences from HCV genotype-1b infected DAA-naïve cirrhotic patients: 34 with HCC and 154 without HCC. Specific NS5A mutations significantly correlate with HCC: S3T (8.8% vs. 1.3%, *p* = 0.01), T122M (8.8% vs. 0.0%, *p* < 0.001), M133I (20.6% vs. 3.9%, *p* < 0.001), and Q181E (11.8% vs. 0.6%, *p* < 0.001). By multivariable analysis, the presence of ≥1 of them independently correlates with HCC (OR (95%CI): 21.8 (5.7–82.3); *p* < 0.001). Focusing on HCC-group, the presence of these mutations correlates with higher viremia (median (IQR): 5.7 (5.4–6.2) log IU/mL vs. 5.3 (4.4–5.6) log IU/mL, *p* = 0.02) and lower ALT (35 (30–71) vs. 83 (48–108) U/L, *p* = 0.004), suggesting a role in enhancing viral fitness without affecting necroinflammation. Notably, these mutations reside in NS5A regions known to interact with cellular proteins crucial for cell-cycle regulation (p53, p85-PIK3, and β-catenin), and introduce additional phosphorylation sites, a phenomenon known to ameliorate NS5A interaction with cellular proteins. Overall, these results provide a focus for further investigations on molecular bases of HCV-mediated oncogenesis. The role of these NS5A domain-1 mutations in triggering pro-oncogenic stimuli that can persist also despite achievement of sustained virological response deserves further investigation.

## 1. Introduction

Hepatocellular carcinoma (HCC) is the fourth leading cause of cancer worldwide [1] causing >600,000 deaths per year [2]. Viral hepatitis B (HBV) and C (HCV) remain the dominant risk factors for HCC. The lifetime risk of developing HCC is 20–30-fold greater for HCV patients than non-infected individuals, and the estimated incidence of HCC in patients with HCV-associated liver cirrhosis and advanced liver fibrosis is about 1–8% per year [3].

HCV-induced HCC is a multifactorial and multistep process involving a combination of cell signaling pathway alterations. Although cirrhosis is the key determinant of HCC in HCV-infected patients [4,5], recent findings have highlighted a direct oncogenic role of the virus [1,5]. Among HCV proteins, NS5A represents one of the central players of liver oncogenesis. NS5A is a 447 amino acid protein, comprises of three domains: domain-1 (residues: 1–213), domain-2 (residues: 250–342), and domain-3 (residues: 356–447) [6]. Notably, NS5A domain-1 is highly conserved among all HCV genotypes compared to domain-2 and -3 [7].

NS5A is required for HCV replication via its association with the endoplasmic reticulum [6] and interaction with different key viral products (NS4B, NS5B, and viral genome) and cellular proteins [6,8]. Additionally, to its role in HCV replication, NS5A protein also contributes to HCV pathogenesis by modulating cell signal pathways and response to alpha-interferon [9]. Moreover, NS5A (particularly domain-1) can interfere with a variety of crucial cellular proteins, such as p53, p85-PI3K, β-catenin, and other different kinases, thus involved in the regulation of cell proliferation and apoptosis [4,10].

HCV genetic variability is also another main determinant of HCC. It has been demonstrated that certain HCV genotypes are associated with a higher risk of HCC. This is the case of genotype-1 and genotype-3 associated with an 80% higher risk of HCC [4]. In line with this observation, HCV genotype-1b, the most prevalent genotype in Italy [11], showed to have a higher risk of developing HCC [2].

Over the past few years, the direct-acting antiviral drugs (DAA) are available for treating chronic HCV patients, with a cure rate of more than 90% [12]. Despite this, HCC can occur even after viral eradication [1,5].

In this light, this study is aimed at spotlighting the existence of genetic determinants within NS5A (focusing on domain-1) associated with HCC in cirrhotic patients with HCV chronic infection, sustained by HCV genotype-1b.

## 2. Materials and Methods

### 2.1. Patients and Samples Collection

This retrospective cohort study included 188 cirrhotic patients chronically infected with HCV genotype-1b monitored from 2013 to 2017 in our hospital (Policlinico Tor Vergata) and different Italian clinical centers. Cirrhosis was recognized by liver-biopsy and/or Fibroscan (>12.5 K Pa), then equalized to Metavir scores. All patients were DAA-naïve: 34 patients diagnosed with HCC and 154 controls patients without HCC. HCC was diagnosed by magnetic resonance imaging or computed tomography. Sample information, together with full clinical and therapy data, were recorded in an anonymous database. HCV-RNA quantification was performed by using Abbott real-time HCV assay (Abbott Laboratories, Abbott park, IL, USA), with a lower limit of detection (LLOD) and quantification (LLOQ) of 12 IU/mL; or COBAS^®^-AmpliPrep/COBAS^®^-TaqMan^®^ HCV Qualitative Test, v2.0 (LLOD = LLOQ = 15 IU/mL; Roche Molecular Systems Inc., Pleasanton, CA, USA).

Approval by the ethics committee was deemed unnecessary under Italian law for all patients evaluated for diagnostic purpose, since this was not considered a clinical trial of medicinal products for clinical use (Art. 6 and Art. 9, Law Decree 211/2003). In the cases evaluated only for research purposes, approval by the local Ethics Committees and patient written informed consent were obtained. The research was conducted on anonymized samples (leg. decree 196/2003).

### 2.2. Population-Based Sequencing of the HCV-NS5A Gene

HCV genome sequencing of the NS5A domain-1 (1–213 amino acids) was performed by using in-house-developed protocols, as previously reported [13] and described in the supplementary method (SM).

### 2.3. Phylogenetic Analysis of NS5A Sequences

Phylogenetic analysis of NS5A sequences by the Tajima-Nei model (MEGA6.1, Hachioji, Tokyo, Japan) was performed to determine the HCV genotype and to exclude potential contaminations (details in SM).

### 2.4. Shannon Entropy Calculation (SE)

Shannon entropy was calculated in order to compare the degree of genetic variability at each NS5A domain-1 position between HCC- and non-HCC patients. Only differences in entropy values ≥ 0.200 with a *p* ≤ 0.05 were considered statistically significant.

### 2.5. Statistical Analysis

Results are expressed as median values and interquartile range (IQR) for continuous data and number (percentage) for categorical data. Categorical variables were compared using the Chi-squared test.

NS5A sequences were used to assess the association of specific NS5A mutations with HCV-related HCC. Mutations were defined according to the reference sequence of each specific HCV genotype (reference sequences: D90208 for genotype 1b). The prevalence of each NS5A mutation was calculated in both HCC and no-HCC patients. Statistically significant differences in the prevalence of NS5A mutations between the two groups of patients were assessed by Chi-squared test.

Factors associated with HCC were evaluated by uni- and multivariable logistic regression analysis, using as confounders: gender, age, HCV-RNA log_10_, liver stiffness, alanine aminotransferase (ALT), aspartate aminotransferase (AST), previous interferon (IFN) usage ± ribavirin, and at least one mutation in NS5A domain-1 associated with HCC. After stepwise elimination for optimized Akaike information criterion, only variables showing a *p*-value ≤ 0.200 in univariable analysis were included in multivariable analysis.

Moreover, we stratified the HCC group according to nodule number and nodule size in order to estimate their correlation with the presence of mutations. For all statistical tests, the level of significance for the evaluation of two-sided *p*-values was set at a 0.05. All the analyses were performed by using the R open-source statistical environment (v.3.3.1) and SPSS software package (v.20) for Windows (SPSS Inc., Chicago, IL, USA).

In order to analyze an independent dataset, HCV genotype 1b NS5A sequences belonging to 140 patients with HCC and 1201 without HCC were retrieved from different publicly databases (GenBank, LosAlamos and European HCV databases, accessed on 1 April 2021). A single sequence for each patient was included in order to avoid duplication. Then, the extent of the genetic variability at each position, associated with HCC in this study, was analyzed and compared in HCC and no-HCC patients.

### 2.6. In-Silico Prediction of the Three-Dimensional NS5A Structure

The three-dimensional (3D) structure NS5A was predicted in-silico using the reference-validated NS5A model [14]. I-TASSER [15] was used to generate the 3D structure of NS5A starting from its amino acid sequence following a homology modelling approach. The fold-stability change (ΔΔG) between wild-type (wt) and mutants was calculated by *STRUM*, with ΔΔG (wt-mutated) value < 0 indicating a reduced stability in the presence of the mutation [16]. Furthermore, the probability of phosphorylation at NS5A by protein kinases was calculated for *wt*-NS5A and mutated-NS5A by prediction algorithm *SCANSITE* [17] aimed at identifying amino acid motifs phosphorylated by Ser/Thr- and Tyr-kinases. *SCANSITE* is well-consolidated and largely-used method for the identification of amino acid motifs phosphorylated by protein Ser/Thr and Tyr kinases. This method is based on a large number of experimental and biochemical data. In particular, in order to determine the substrate specificity of protein kinases, Scansite uses a library containing more than 2.5 billion peptides (with a central residue of Ser, Thr, or Tyr). These peptides are incubated with specific Ser/Thr and Tyr kinases and the efficiency of phosphorylation (measured by the amount of radioactive phosphate bound to the peptide) is then elaborated by using advanced machine learning approaches (including Markov models or artificial neural networks) and expressed as numerical scores.

## 3. Results

### 3.1. Patients’ Characteristics

The NS5A domain-1 was successfully amplified and sequenced for all 188 patients. The clinical and virological characteristics of patients are shown in Table 1. Patients had a median (IQR) age of 70 (60–75) years, 54.8% were male and the majority of them were Italian (177/188, 94%).

All patients were DAA-naïve, and the 65% of them had previous exposure to alpha-interferon. Patients with HBV and HIV coinfection were 3.7% (7/188) and 1.1% (2/188), respectively. No differences were observed for HCV-RNA and ALT between HCC and no-HCC patients (HCV-RNA: 5.6 (5.3–6.1) vs. 5.8 (5.3–6.1) log IU/mL; ALT: 65 (37–86) vs. 71 (50–112) U/L) (Table 1). Conversely, HCC patients had significantly higher liver stiffness than no-HCC patients (28 (20–33) vs. 19 (15–26) K Pa, *p* < 0.001) (Table 1).

### 3.2. Association of Mutations in NS5A Domain-1 with HCV-Related HCC

**Figure 1:**
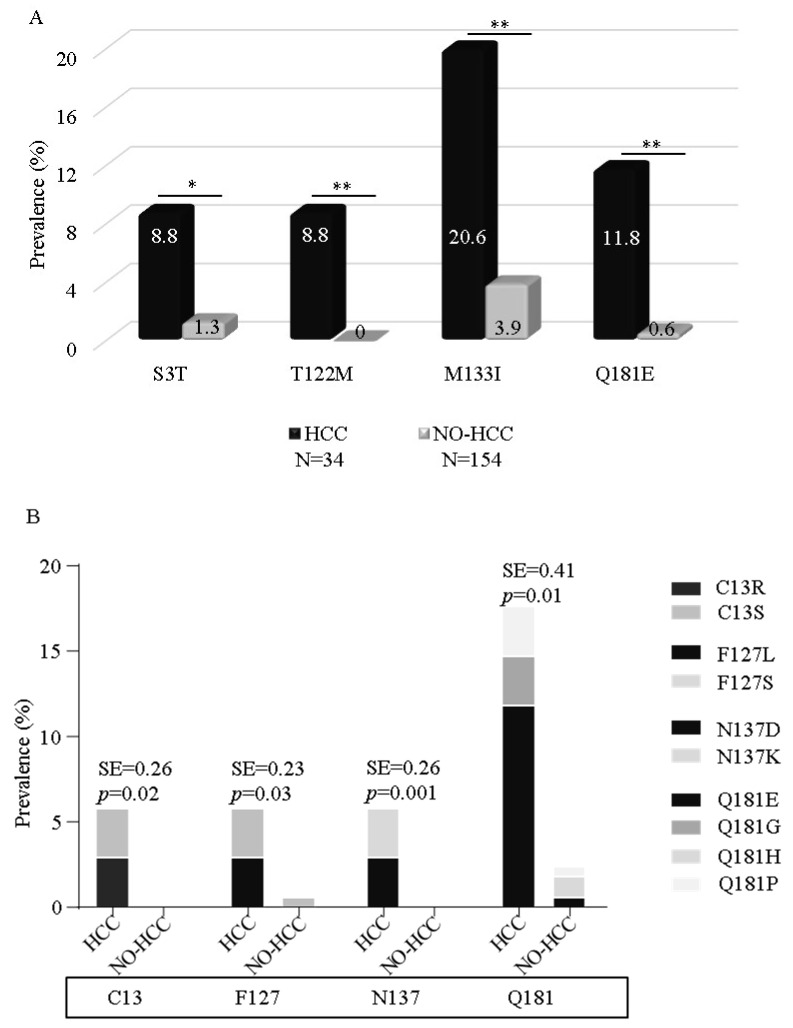
Prevalence of NS5A mutations in patients with or without HCC. (**A**) The histogram reports the prevalence of NS5A domain-1 mutations in HCC cirrhotic patients (*N* = 34), and no-HCC cirrhotic patients (*N* = 154). Only mutations significantly correlated with HCC are reported. Statistically significant differences were assessed by using the Fisher exact test. * *p* < 0.01, ** *p* < 0.001. (**B**) The histogram reports the prevalence of patients with mutations at residues 13, 127, 137, and 181 in HCC and no-HCC patients. The abovementioned residues had significantly higher Shannon entropy values in HCC than in no-HCC patients. Shannon entropy was calculated by submitting NS5A domain-1 amino acid sequences to HCV Los Alamos National Laboratory (LANL) Entropy-Two tool (https://hcv.lanl.gov/content/sequence/ENTROPY/entropy.html, accessed on 3 February 2021). Only differences in entropy values ≥ 0.2 with a *p* ≤ 0.05 were considered statistically significant.

The mutational analysis revealed four specific NS5A domain-1 mutations significantly correlated with HCC (Figure 1A). Indeed, their prevalence showed a significant increase in HCC- compared to no-HCC patients: S3T (8.8 vs. 1.3%, *p* = 0.01), T122M (8.8% vs. 0.0%, *p* < 0.001), M133I (20.6 vs. 3.9%, *p* < 0.001), and Q181E (11.8 vs. 0.6%, *p* < 0.001). Interestingly, about half of the HCC patients (16/34, 47.1%) carried at least one of these mutations compared to only 5.8% (9/154) of no-HCC ones. Multivariable analysis confirmed the independent association of at least one mutation in NS5A domain-1 with a higher probability of developing HCC (adjusted OR (95%CI): 21.8 (5.7–82.3); *p* < 0.001), after correction for patients’ confounders (Table 2). As expected, other factors independently associated with HCC were sex (8.5 (2.0–35.9), *p* = 0.003) and liver stiffness (1.1 (1.0–1.1), *p* = 0.011).

Interestingly, by Shannon entropy analysis, other three residues in NS5A domain-1 showed a higher degree of genetic variability in HCC than in no-HCC patients (Figure 1B): This is the case of residue 13 (SE: 0.26 in HCC vs. 0.00 in no-HCC *p* = 0.02), residue 127 (SE: 0.26 in HCC vs. 0.04 in no-HCC, *p* = 0.03), and residue 137 (0.26 in HCC vs. 0.00 in no-HCC, *p* = 0.001). Furthermore, an enrichment of amino acid mutations was also observed at residue 181 (SE: 0.56 vs. 0.15; *p* = 0.01) (Figure 1B).

By analyzing an independent dataset of NS5A genotype 1b sequences retrieved from available databases (140 for HCC and 1201 for no-HCC group), the presence of at least one mutation at the abovementioned positions was significantly higher in HCC than in no-HCC patients (20% vs. 2.4%, *p* < 0.001). This is particularly evident for position 3 in which the prevalence of NS5A sequences with at least one mutation was significantly higher in HCC than in no-HCC patients (3.6% vs. 0.6%, *p* < 0.001).

### 3.3. Association of NS5A Domain-1 Mutations with Virological and Biochemical Parameters

The next step of this study was to investigate in HCC-group the correlation of the mutations (S3T, T122M, M133I, and Q181E) with serum HCV-RNA and transaminases. Interestingly, the presence of ≥1 mutation associated with HCC was significantly correlated with higher serum HCV-RNA (5.7 (5.4–6.2) log IU/mL vs. 5.3 (4.4–5.6) log IU/mL, *p* = 0.02) (Supplementary Materials Figure S1), suggesting a potential impact of these mutations in enhancing viral fitness. Conversely, a negative correlation was found with transaminases (median (IQR) ALT: 35 (30–71) vs. 83 (48–108) U/L, *p* = 0.004 and median (IQR) AST: 39 (30–71) vs. 84 (59–119) U/L, *p* = 0.01), suggesting that the involvement of these mutations in HCC onset may not be mediated by enhancing necroinflammation. No significant correlations were found between the presence of ≥1 mutation associated with HCC and the following parameters: time since HCV diagnosis, previous use of IFN and/or Ribavirin, α-fetoprotein levels, and number and size of nodules.

### 3.4. Localization of Mutations in Functional Regions of NS5A Domain-1 and Their Impact on the Three-Dimensional Structure of NS5A

**Figure 2:**
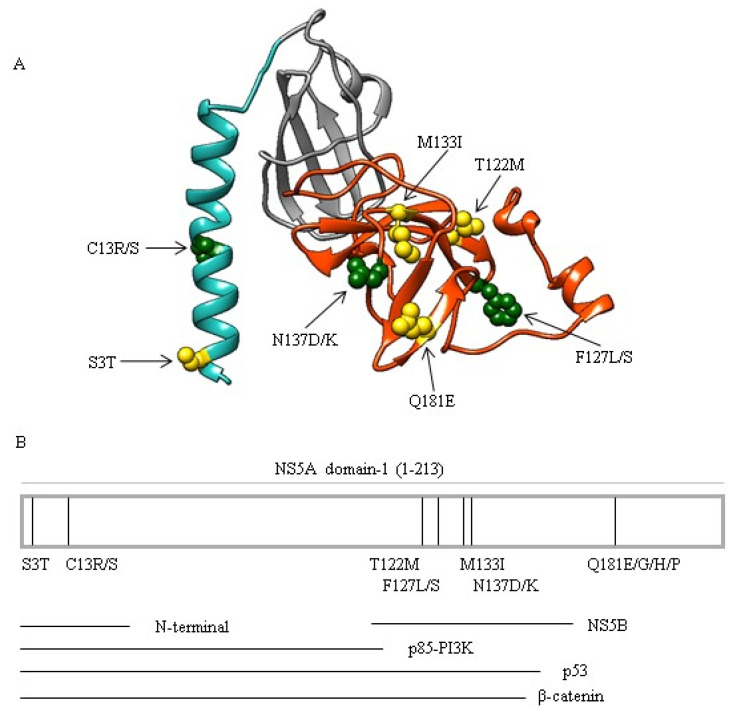
Localization of mutations in NS5A. (**A**) Three-dimensional structure of the NS5A domain-1 showing the localization of mutations associated with HCC. Cyan alpha helix refers to N-terminal NS5A, grey beta-sheets refer to zinc binding domain, while orange beta-sheets refer to NS5A domains involved in binding to NS5B and viral RNA genome. The four mutations associated with HCC are highlighted in yellow, while the three residues with significantly higher variability in the HCC group are highlighted in green. (**B**) Schematic representation of the NS5A domain-1 (213 amino acids) showing the localization of the mutations associated with HCC in NS5A domain-1 region involved in the interactions with the cellular proteins (p53, p85-PI3K, and β-catenin), and with the viral protein NS5B.

By in-silico prediction of three-dimensional NS5A structure, the mutations at positions 3 and 13 lie in the α-helices of the NS5A N-terminus known to mediate the anchorage of NS5A in the membrane of the endoplasmic reticulum [18]. Conversely, mutations at positions 122, 127, and 133 and 137 lie in a β-sheet-enriched region (aa: 105–162) known to directly interact with the viral protein NS5B [19]. Furthermore, the mutations at position 181 lie in a β-sheet of the NS5A domain-1 C-terminus known to act as RNA binding subdomain (aa: 101–213) and to have an important role in HCV replication cycle [20] (Figure 2).

Remarkably, all the mutations associated with HCC are localized in regions of NS5A domain-1 interacting with cellular proteins such as p53, p85-PIK3, and β-catenin, pivotal in the regulation of cell growth and apoptosis (Figure 2B).

Recent studies have highlighted that the phosphorylation of NS5A is a key post-translational modification important to modulate NS5A functions [21,22]. Interestingly, by applying an in-silico prediction model, specific mutations associated with HCC introduced novel sites for phosphorylation by kinases, including the above-mentioned p85-PIK3 and other kinases involved in cell-cycle regulation (Table 3).

Finally, the impact of mutations associated with HCC on NS5A structural stability was investigated. Most of them did not determine significant changes in the stability of NS5A with the exception of the two mutations at position 13 and F127S causing a reduction of −1.74, −1.86, and −1.08 Kcal/mol in NS5A stability compared to wt (Supplementary Materials Table S1).

## 4. Discussion

This study has identified specific NS5A domain-1 genetic elements significantly correlated with HCC, in cirrhotic patients with HCV chronic infection, sustained by HCV genotype-1b. Previous studies have investigated the involvement of mutations in HCC onset primarily in core protein [23], NS5A domain-2 [24], and NS5A domain-3 [25]. Although in-vitro studies are needed, to the best of our knowledge, this is the first study addressing this issue on NS5A domain-1.

In particular, four specific mutations were found significantly associated with HCC (S3T, T122M, M133I, and Q181E). Notably, about half of the HCC patients had at least one of them and the multivariable logistic regression analysis confirmed their independent association with HCC.

Beyond the above-mentioned mutations, Shannon entropy analysis identified specific residues in NS5A domain-1 characterized by a significant higher degree of genetic variability in HCC than in the no-HCC group, suggesting that mutations at these positions arise following a process of positive selective pressure rather than random genetic drift.

Notably, by analyzing an independent dataset of NS5A genotype 1b sequences retrieved from available databases (140 for HCC and 1201 for no-HCC group), the presence of at least one mutation at the abovementioned positions was significantly higher in HCC than in no-HCC patients (20% vs. 2.4%, *p* < 0.001), supporting the role of an enriched genetic variability at these positions in HCV oncogenic potential.

Furthermore, by analyzing another set of NS5A sequences from 184 non-cirrhotic, non-HCC and DAA-naïve HCV genotype-1b infected patients, these mutations were absent/nearly absent (0% F127L/S and N137D/K; ≤1% C13S, T122M and Q181E) or detected with a frequency never exceeding 4% (S3T and M133I), excluding the polymorphic nature of these mutations. Such frequencies were significantly lower than those observed in HCC HCV genotype-1b infected patients, further supporting the association of these mutations with HCC.

Finally, in a dataset of NS5A sequences specifically isolated from plasma, tumoral, and peritumoral liver tissue samples of seven cirrhotic HCV genotype-1b infected patients with HCC [26], ≥1 mutation associated with HCC was detected in 43% (3/7) in all the three compartments. Furthermore, an enrichment of amino acid mutations at positions 133 and 181 was also observed in two other patients, again in all the compartments analyzed.

The association of mutations in NS5A domain-1 with HCC can be potentially explained by their localization in regions known to interact not only with viral components but also with a variety of cellular proteins. Notably, all the identified mutations reside in regions known to directly interact with cellular proteins critical for the regulation of cell-cycle including p53, p85-PIK3, and β-catenin [10]. In particular, previous studies have shown that the first 150 residues of NS5A domain-1 directly bind and sequester p53 in the cytoplasmic/perinuclear regions, thus inhibiting p53-induced apoptosis [27]. Similarly, the N-terminus of NS5A can also directly interact with β-catenin thus inhibiting TNF-alpha-induced apoptosis [10,28]. Furthermore, the first 110 residues of NS5A domain-1 bind p85-PI3K, thus activating Akt and Wnt/β-catenin signaling pathways known to act as key regulators of cell proliferation [10,28]. Although in vitro studies are necessary, it is plausible to hypothesize that these mutations might enhance NS5A capability to interact with these crucial regulators of cell-cycle. This in turn could inhibit apoptosis and/or promote cell proliferation, thus posing the basis for the neoplastic transformation of the hepatocytes [29].

In line with this concept, by in-silico prediction analysis, some of the identified mutations can increase the probability of phosphorylation of NS5A domain-1 by several kinases. Remarkably, a recent study demonstrated that phosphorylation of Y93 in NS5A plays a crucial role in facilitating NS5A interaction with the proto-oncogene c-Src kinase [22]. Further studies, analyzing NS5A domains other than domain-1, demonstrated that a reduced phosphorylation at residue 225 can hinder viral genome replication and NS5A interactions with key cellular proteins [21]. Similarly, an impaired phosphorylation at residues P351 and P354 can hamper the interaction with MLK3 thus inducing apoptosis [30]. Furthermore, abrogating the phosphorylation at residues 353–355 impaired antiapoptotic activity of NS5A [31].

Based on these findings, the enrichment of phosphorylation sites, associated with the identified mutations, could be a potential mechanism by which mutations associated with HCC, could exert their anti-apoptotic and/or pro-oncogenetic activities, thus potentially contributing along with cirrhosis to the neoplastic transformation of the hepatocytes. Further in vitro studies are necessary to finely unravel this issue.

This study also shows that the presence of at least one mutation associated with HCC correlates with significantly higher serum HCV-RNA and lower transaminases, suggesting a role of these mutations in promoting viral fitness without affecting necroinflammation process. In particular, mutations at residues 122, 127, 133, and 137 are localized in a region (residues: 105–162) critical for the binding to NS5B [8], and in turn for RNA replication [19], suggesting their role in enhancing NS5A capability to interact with NS5B and in turn to promote viral fitness.

Overall findings highlight a dual role of these mutations in enhancing viral fitness and in promoting hepatocyte survival, thus allowing long-term production of infectious progeny. At the same time, this may pose the basis for clonal selection of hepatocytes harboring these viral variants, favoring the development of liver cancer.

Different studies have highlighted that the risk to develop HCC (de novo or recurrence) can persist (even if attenuated) after successful treatment with DAAs [1,5,32] particularly in cirrhotic patients. This can be due also to the persistence of pro-oncogenic stimuli even after the achievement of sustained virological response [1,5]. In this light, a further investigation on molecular bases of NS5A mutants and HCV-mediated oncogenesis deserves attention, and the investigation on clinical follow-up of cirrhotic HCV genotype-1b infected patients harboring these mutations. These mutations enhancing NS5A capability to interact with cellular proteins crucial in cell-cycle regulation, could act as predictive markers of HCC, helping to identify cirrhotic patients at higher HCC risk, who need a closer monitoring even after eradicating treatment.

This study was focused on HCV genotype-1b, it would be useful to verify if the genetic backbone of other HCV genotypes can favor the emergence of these mutations and in turn their involvement in HCC onset. At this regard we retrieved from our and publicly available databases 1187 NS5A genotype 3 sequences from 14 patients with HCC and 1173 without HCC. The percentage of at least one mutation associated with HCC showed no statistically significant difference between the two groups of patients (42.9% vs. 35.8%, *p* = 0.6), suggesting that these mutations might not play a role in the setting of genotype 3. Furthermore, it would be interesting to verify the synergistic role of HBV infection in enhancing HCV pro-oncogenic properties.

In conclusion, specific mutations in NS5A domain-1 significantly correlate with HCV genotype-1b induced HCC, presumably by enhancing NS5A capability to interact with cellular proteins crucial in cell-cycle regulation. The role of these mutations in triggering persistent pro-oncogenic stimuli even after the achievement of sustained virological response deserves further investigation in in-vitro studies.

## Figures and Tables

**Table 1:** Characteristics of the study population (*N* = 188).

Number of Patients	Overall (*N* = 188)	HCC (*N* = 34)	No-HCC (*N* = 154)	*p*-Value ^a^
Male, *n* (%)	103 (54.8)	26 (76.5)	77 (50.0)	0.007
Median (IQR) Age (Years)	70 (60–75)	75 (68–78)	68 (60–74)	0.012
Italian Nationality, *n* (%)	177 (94)	34 (100)	143 (93)	0.075
Cirrhotic, *n* (%)	188 (100)	34 (100)	154 (100)	-
Median (IQR) Liver Stiffness (K Pa)	20 (15.1–27.7)	28 (20.0–33.0)	19 (15.0–26.3)	<0.001
Median (IQR) HCV-RNA (log IU/mL) ^b^	5.8 (5.3–6.1)	5.6 (5.3–6.1)	5.8 (5.3–6.1)	0.350
Median (IQR) ALT (IU/L)	69 (46–106)	65 (37–86)	71 (50–112)	0.126
Median (IQR) AST (IU/L)	71 (46–110)	64 (38–100)	75 (46–110)	0.230
Median (IQR) year of first HCV positivity	1999 (1994–2006)	2001 (1993–2002)	1999 (1994–2006)	<0.001
***Risk factor, n (%)***				
Drug usage	7 (3.7)	1 (2.9)	6 (3.9)	0.071
Parental	4 (2.1)	2 (5.9)	2 (1.3)	0.094
Transfusion	25 (13.3)	5 (14.7)	20 (13.0)	0.071
Iatrogenic	12 (6.4)	6 (17.6)	6 (3.9)	0.002
Sexual	14 (7.5)	1 (2.9)	13 (8.4)	0.269
Professional exposure	1 (0.5)	1 (2.9)	0 (0.0)	0.011
Unknown	125 (66.5)	18 (52.9)	107 (69.5)	-
***Therapeutic Information, n (%)***				
Naive to DAA treatment	188 (100)	34 (100)	154 (100)	-
Previous IFN Failure	62 (65)	13 (59.1)	49 (67.1)	0.488
***HCC characteristics***				
Single nodule HCC, *n* (%)	-	8 (23.5)	-	-
Multi-focal HCC, *n* (%)	-	9 (26.5)	-	-
Range from plasma sample collection to HCC diagnosis, Months	-	(−20–1)	-	-
Median (IQR) α-fetoprotein at HCC diagnosis (ng/mL)	-	23.5 (7.9–34.2)	-	-

^a^ By Mann Whitney or Chi-squared test. ^b^ HCV-RNA values are the closest or concomitant to the sequencing of NS5A. Abbreviations: ALT, alanine aminotransferase; AST, aspartate aminotransferase; IFN, interferon; DAA, direct-acting antiviral; HCV, hepatitis C virus; HCC, hepatocellular carcinoma; IQR, interquartile range; hyphen, unapplicable data.

**Table 2:** Factors associated with HCV-related HCC by multivariable logistic regression analysis.

Variables ^a^	Univariable Analysis		Multivariable Analysis	
	Crude OR (95% CI)	*p*-Value	Adjusted OR (95% CI)	*p*-Value
Gender (male vs. female)	5.3 (1.7–16.3)	**0.003**	8.5 (2.0–35.9)	**0.003**
Age (for 1 year increase)	1.0 (1.0–1.1)	0.088	1.0 (1.0–1.1)	0.152
Liver Stiffness, K Pa	1.0 (1.0–1.1)	**0.009**	1.1 (1.0–1.1)	**0.011**
HCV-RNA, log_10_ IU/mL	0.9 (0.6–1.4)	0.645	-	-
ALT, U/L	1.0 (1.0–1.0)	0.297	-	-
AST, U/L	1.0 (1.0–1.0)	0.284	-	-
Previous IFN usage ± ribavirin	1.8 (0.8–4.2)	0.176	2.5 (0.8–8.3)	0.126
At least one mutation in NS5A-domain-1	18.8 (6.2–56.3)	**<0.001**	21.8 (5.7–82.3)	**<0.001**

^a^ The logistic regression analysis was performed on 140 chronically HCV-infected patients with or without HCC. The following variables were considered: gender, age, HCV-RNA log_10_, liver stiffness, ALT, AST, previous IFN usage ± ribavirin, and at least one mutation in NS5A domain-1 associated with HCC (S3T, T122M, M133I, and Q181E). Only variables showing a *p*-value ≤ 0.200 in univariable analysis were included in multivariable analysis. Abbreviations: ALT, alanine aminotransferase; AST, aspartate aminotransferase; IFN, interferon; HCV, hepatitis C virus; HCC, hepatocellular carcinoma; OR, odds ratio; CI, confidence interval. Statistically significance *p*-value (<0.05) were reported in Bold.

**Table 3:** NS5A domain-1 mutations associated with the acquisition of novel phosphorylation sites by the in silico prediction model.

Mutation	Position	Potential Kinase	Phosphorylation Score ^a^ in Wild-Type/Mutant
C13R	T14	NEK10	0.000/0.923
	T14	NEK3	0.000/1.008
	T14	NEK5	0.000/0.895
	T14	NEK8	0.000/1.003
C13S	S13	GSK3	0.000/0.745
	S13	NEK8	0.000/0.960
F127S	T122	PDPK1	0.000/1.026
	Y129	PDGF-R	0.000/0.575
	Y129	p85-PI3K	0.000/0.767
N137K	T134	CDK1	0.000/1.037
	T134	PKC	0.000/0.596
	T135	NEK4	0.000/0.956

^a^ The score was calculated by SCANSITE and measures the probability of phosphorylation at given position. The residue is predicted to be phosphorylated, because the score is above the threshold of 0.500 (value in the range (0.000–1.400)). Abbreviations: NEK, NIMA-related kinase; GSK3, glycogen synthase kinase *3*; PDPK1, 3-phosphoinositide-dependent protein kinase-1; PDGF-R, platelet-derived growth factor receptors; PI3K, phosphoinositide 3-kinases; CDK, cyclin-dependent kinases; PKC, protein kinase C.

## Data Availability

The data presented in this study are available on request from the corresponding author.

## Supplementary Materials

The following are available online at https://www.mdpi.com/article/10.3390/v13050743/s1, Supplementary materials, Figure S1: Association of NS5A mutations with HCV viremia, Table S1: Impact of NS5A Mutations associated with HCC on protein stability and their localization.
