# Supplementary material for: Genetic Determinants in a Critical Domain of NS5A Correlate with Hepatocellular Carcinoma in Cirrhotic Patients Infected with HCV Genotype 1b
Source: Viruses. 2021 Apr 23;13(5):743. doi: 10.3390/v13050743 (PMC8146897; doi:10.3390/v13050743)
Supplement: Supplementary file 1 [file viruses-13-00743-s001.zip › viruses-1153216-supplementary/Supplementary Figure S1.pdf]

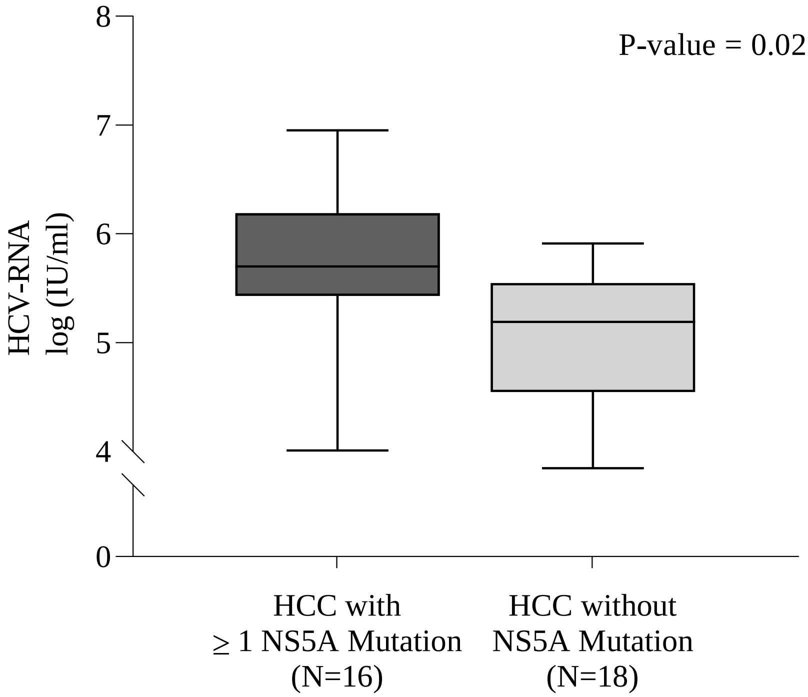

Figure S1. Association of NS5A mutations with HCV viremia.

The boxplot reports the distribution of HCV-RNA values in HCC patients with at least one mutation associated with HCC (N=16) and in HCC patients without these mutations (N=18). Statistically significant difference was assessed by using Mann-Whitney test.
