# Supplementary material for: Genetic Determinants in a Critical Domain of NS5A Correlate with Hepatocellular Carcinoma in Cirrhotic Patients Infected with HCV Genotype 1b
Source: Viruses. 2021 Apr 23;13(5):743. doi: 10.3390/v13050743 (PMC8146897; doi:10.3390/v13050743)
Supplement: Supplementary file 1 [file viruses-13-00743-s001.zip › viruses-1153216-supplementary/Supplementary Matirals.pdf]

Supplementary Methods

Sanger sequencing of the HCV-NS5A gene

HCV genome sequencing of the NS5A domain-1 (1-213 amino acids) was performed by using in-house-developed protocols, specifically designed for subtype 1b (Di Maio *et al.*, 2017). HCV-RNA was extracted using a standard commercial silica gel membrane-binding method (QIAamp Viral RNA Mini Kit; Qiagen, Valencia, CA, USA). According to viremia levels, for samples with an HCV-RNA 6 log IU/mL, 1 mL of plasma was before concentrated by ultracentrifugation at 25000 g for 1.30 h at 4 °C, while for samples with an HCV-RNA ≥6 log IU/mL,140 mL of plasma was directly used to perform RNA extraction. Synthesis and amplification of cDNA were performed in a single step by using the commercial SuperScript III One-Step RT–PCR System with Platinum Taq High Fidelity (Invitrogen, Carlsbad, CA, USA) and specific primers designed for the HCV-1b subtype (Di Maio *et al.*, 2017).

The first-round PCR was performed as follows: 10 μL of RNA was added to 40 μL of PCR mixture containing 25 μL of (2×) reaction buffer, 8 μL of 5 mM Mg^{2+}, 3 μL of DNase/RNase-free water, 1 μL of each primer at a concentration of 10 μM, 1 μL of RNase OUT (40 U/μL) and 1 μL of reverse transcriptase/Taq. Reverse transcription was performed for 30 min at 45 °C, followed by denaturation for 2 min at 94 °C and amplification over 40 cycles at 94 °C for 30 s, (57 °C for amplicon) for 30 s, 68 °C for 90 s and a final 10 min extension step at 68 °C. The amplified product was run on a 1% agarose gel. If necessary, a nested PCR was also performed.

Nested-PCR protocols was performed as follows: 5 μL of amplified product was denatured at 94 °C for 12 min and amplified with 35 cycles at 94 °C for 30 s, (56 °C for amplicon) for 30 s and 68 °C for 90 s, by using the following reaction mixture with Ampli taq Gold DNA polymerase: 5 μL of 10× Taq buffer, 4 μL of 25mM Mg^{2+}, 32.5 μL of DNase/RNase-free water, 0.9 μL of each primer at a concentration of 10 mM, 1 μL of 10 mM deoxynucleotides and 0.7 μL of Taq (5 U/mL) for a total of 45 μL.

Finally, NS5A amplified products were sequenced by an automated sequencer (ABI-3130) in sense and antisense orientation using four different overlapping sequence-specific primers for each amplicon using the Big-Dye Terminator v.3.1 Cycle Sequencing Kit (Applied Biosystems). Sequences having a mixture of wild-type and mutant residues at a single position were considered to have the mutant(s) at that position.

Furthermore, in our standardized sequencing procedure, positive control plasma samples were reprocessed over time. Complete concordance in the detection of mutations between the repeated sequences was always found.

Phylogenetic analysis of NS5A sequences

Phylogenetic analysis of NS5A sequences by the Tajima-Nei model (MEGA6.1) was performed to determine HCV genotype, test sequencing reliability and the possibility of cross-contamination or sample mix-up during laboratory procedures and to evaluate concordance with previous subtype assignment by commercial genotyping assays. NS5A sequences were aligned using the Clustal W algorithm integrated into BioEdit software. Then, all sequences were compared with the reference strain of genotypes 1b (GenBank accession number: HCV-1b, D90208) using the neighbor-joining method 38 and the Kimura two-parameter distance estimation approach 39 in MEGA v5.1.40. The reliability of the phylogenetic clustering was assessed by bootstrap analysis of 1000 replicates, later then the aligned sequences were all compared to the reference strain in SeqScape v2.5 software to analyze the mutations at each amino acid position in NS5A domain-1.

Shannon Entropy calculation (SE)

The analysis was performed by comparing the entropy values of HCC and No-HCC groups to objectively evaluate the variability of each amino acid position in NS5A domain-1. Amino acid sequences were submitted to the HCV Los Alamos National Laboratory (LANL) Entropy-Two tool (https://hcv.lanl.gov/content/sequence/ENTROPY/entropy.html). Only the entropy differences between 2 sets ≥ 0.200 followed by a $P \leq 0.05$ were considered statistically significant.
