# Supplementary material for: Genetic Determinants in a Critical Domain of NS5A Correlate with Hepatocellular Carcinoma in Cirrhotic Patients Infected with HCV Genotype 1b
Source: Viruses. 2021 Apr 23;13(5):743. doi: 10.3390/v13050743 (PMC8146897; doi:10.3390/v13050743)
Supplement: Supplementary file 1 [file viruses-13-00743-s001.zip › viruses-1153216-supplementary/Supplementary Table S1.pdf]

**Table S1. Impact of NS5A Mutations associated with HCC on protein stability and their localization.**

| # | Mutation | ΔΔG[a] | Location in NS5A | | Interacting Protein/Nucleic Acid |
|---|---|---|---|---|---|
| 1 | S3T | -0.36 | Alpha-helix | N-terminal | p53, p85-PI3K, β-catenin |
| 2 | **C13S** | **-1.86** | | | |
| 3 | **C13R** | **-1.74** | | | |
| 4 | T122M | 0.06 | Beta-sheet | Domain-1 | NS5B, p53, p85-PI3K, β-catenin |
| 5 | F127L | -0.43 | | | |
| 6 | **F127S** | **-1.08** | | | |
| 7 | M133I | -0.31 | | | |
| 8 | N137D | 0.54 | Coil-turn | | |
| 9 | N137K | 0.16 | | | |
| 10 | Q181E | 0.62 | Beta-sheet | | Viral RNA genome |
| 11 | Q181G | 0.52 | | | |
| 12 | Q181H | 0.53 | | | |
| 13 | Q181P | 0.22 | | | |

[a] ΔΔG (kcal/ mol) = (ΔG wt fold - ΔG mutant fold) was calculated by STRUM algorithm. A negative ΔΔG value indicates a decrease in protein stability.

The mutations with ΔΔG < -1.0, indicating a relevant decrease in protein stability is reported in bold
